# Inflammatory responses to a pathogenic West Nile virus strain

**DOI:** 10.1186/s12879-019-4471-8

**Published:** 2019-10-29

**Authors:** Bixing Huang, Nic West, Jelena Vider, Ping Zhang, Rebecca E. Griffiths, Ernst Wolvetang, Peter Burtonclay, David Warrilow

**Affiliations:** 1 Public Health Virology Laboratory, Queensland Health Forensic and Scientific Services, PO Box 594, Archerfield, Queensland Australia; 2 Menzies Health Institute Queensland and School of Medical Science, Griffith University, Southport, Queensland Australia; 3 Australian Institute for Bioengineering and Nanotechnology, University of Queensland, Brisbane, Queensland Australia

**Keywords:** Flavivirus, Encephalitis, Arbovirus, Innate immunity, Stem cell, Inflammation

## Abstract

**Background:**

West Nile virus (WNV) circulates across Australia and was referred to historically as Kunjin virus (WNV_KUN_). WNV_KUN_ has been considered more benign than other WNV strains circulating globally. In 2011, a more virulent form of the virus emerged during an outbreak of equine arboviral disease in Australia.

**Methods:**

To better understand the emergence of this virulent phenotype and the mechanism by which pathogenicity is manifested in its host, cells were infected with either the virulent strain (NSW2012), or less pathogenic historical isolates, and their innate immune responses compared by digital immune gene expression profiling. Two different cell systems were used: a neuroblastoma cell line (SK-N-SH cells) and neuronal cells derived from induced pluripotent stem cells (iPSCs).

**Results:**

Significant innate immune gene induction was observed in both systems. The NSW2012 isolate induced higher gene expression of two genes (IL-8 and CCL2) when compared with cells infected with less pathogenic isolates. Pathway analysis of induced inflammation-associated genes also indicated generally higher activation in infected NSW2012 cells. However, this differential response was not paralleled in the neuronal cultures.

**Conclusion:**

NSW2012 may have unique genetic characteristics which contributed to the outbreak. The data herein is consistent with the possibility that the virulence of NSW2012 is underpinned by increased induction of inflammatory genes.

## Background

West Nile virus (WNV) is a globally-distributed mosquito-borne virus. In Australia, it was first isolated from *Culex annulirostris* mosquitoes in northern Queensland in 1960 [1], and has most likely been part of the country’s ecology for a considerable period of time. Historically, it was referred to as Kunjin virus, but was re-classified as a subtype of West Nile virus (WNV_KUN_) [2, 3]. WNV_KUN_ can cause disease including encephalitis in human and horses, but is generally considered more benign than other WNV strains circulating globally [2]. WNV_KUN_ is genetically homogenous across Australia due to the nature of its repeated dissemination from foci located in the tropical north of the country to southern interior regions by migrating wading birds (e.g. egrets and herons) after flooding rains [4, 5].

In 2011 there was a serious outbreak of equine arboviral disease in Australia in approximately 1000 horses, resulting in neurological disease and death in 10% of cases [6]. WNV_KUN_ was the agent predominantly responsible. Despite the circulation of this virulent strain of WNV_KUN_, there was only a single human case around the time of the outbreak [7]. The drivers of the emergence of this more virulent form of the disease are not entirely clear, are likely to be multi-factorial and include viral fitness for its *Cx. annulirostris* vector [8], and possibly other environmental factors. The WNV_KUN_ strain circulating at the time of the outbreak showed increased neurovirulence in mice relative to historically circulating strains [7]. Sequencing revealed the presence of virulence markers (a glycosylation site at amino acid 154 of E and a phenylalanine at 653 of NS5) [5, 7]. However, these were also present in older less virulent strains and, therefore, could not be solely responsible for the increased virulence. Hence, other unknown genome changes are likely to have contributed to the disease outbreak.

Symptomatic flavivirus infection is generally believed to have an immune-mediated component (reviewed in [9–12]). Infection results in activation of innate and adaptive immune responses which attempt to control infection, but may also contribute to pathology, particularly in the case of encephalitis. After cell entry, virus RNA and proteins may be recognized as a pathogen-associated molecular pattern (PAMP) by host pattern recognition receptors (PRRs) such as cGAS, RIG-1 and TLR3/7 (reviewed in [13, 14]). These activate signaling pathways resulting in IFNα/β production, and subsequent autocrine and paracrine activation of interferon stimulated genes (ISGs) through the JAK-STAT pathway. ISGs such as viperin, IFIT2, OAS/RNaseL and PKR have effector function in vivo which can control WNV replication. The efficaciousness of these ISGs is often tissue dependent; therefore, the experimental system chosen to study these responses is critical.

We performed this study on innate responses to WNV_KUN_ for three reasons. Firstly, it was an opportunity to study the emergence of a more virulent virus strain. Secondly, to consider the respective contributions of virus genetics, host immune response, and the interplay between them, to disease pathogenesis. Thirdly, a better understanding of this process may lead to the development of a diagnostic assay, the basis of which is the detection of a unique host innate immune gene induction profile which reflects the virulence of a newly isolated circulating strain. The latter is a longer-term objective, which would act as a valuable early warning system in the early phase of virus emergence. Hence, it was necessary to develop an assay using a relevant cell system. In pursuing this objective, we explored two different cell systems. One was the human neuroblastoma cell line SK-N-SH, and the other was a neuronal culture derived from human induced pluripotent stem cells (iPSCs). We measured the induction of a panel of 249 inflammation-associated genes after infection with WNV_KUN_ isolates. Gene induction varied with the isolate and the cell systems used, and revealed at least two host markers induced by infection with a more virulent virus.

## Methods

### Cell culture and virus infections

The human neuroblastoma cell line SK-N-SH was grown in Dulbecco’s Modified Eagles Medium (DMEM) with fetal calf serum (10% vol./vol.) and incubated at 37°C in 6-well plates. The cells were induced to differentiate 72 h prior to experimental work by the addition of retinoic acid (5 μM) to the medium. Cerebral organoids were generated from human induced pluripotent stem cells (iPSC) from a single donor as previously described (Lancaster et al., 2013). After neural induction, the organoids were dissociated in Accutase (StemCell Technologies) and plated onto poly-O-ornithine (Sigma-Aldrich) and laminin (Sigma-Aldrich) coated plates. After plating neuronal differentiation (ND) media was used to feed the cells every other day. ND media comprised 1:1 DMEM/F12 (Gibco) and Neurobasal Media 1% w/v Glutamax (Gibco), 1% w/v MEM–NEAA (Gibco), 1% w/v 10,000 U/mL penicillin–streptomycin (Gibco), 1% w/v N2, 2% w/v B27 (Life Technologies) and 0.02% w/v insulin (Sigma-Aldrich) with 20 ng/ml GDNF, 20 ng/mL BDNF (Life Technologies), 0.8ug/ml laminin, 0.2 μM ascorbic acid and 500 μM dbcAMP (Sigma-Aldrich).

All live virus work was conducted in a certified physical containment level 3 (BSL-3 equivalent) laboratory under 50 Pa pressure differential using a class 2 biosafety cabinet whilst wearing appropriate personal protective equipment. For cell infections, virus was added at the multiplicity of infection of 0.1. The cells were then incubated for 4 h before the medium was aspirated, the cells washed twice with DMEM, and fresh growth medium added before further incubation for 48 h. Immuno-stimulators LPS (100 ng/ml; 24 hour induction) and poly(I:C) (20 μg/ml; 24 hour induction) were included as controls for immune gene induction. To prepare cells for harvest, the growth medium was removed, the cells were washed once in sterile PBS, 0.1 mM EDTA was added, and the cells incubated at 37°C for 10 min. The cells were resuspended from the plate surface by pipetting, centrifuged (14,000 RPM in a benchtop centrifuged), the supernatant aspirated, and the pellet was stored at -80°C. RNA was extracted using a RNAeasy kit (Qiagen). Quantitative reverse-transcription PCR (qRT-PCR) to detect virus infection [15] was used to confirm successful infection by comparing threshold cycle values (C_T_) of day 0 and 2 post-infection supernatant samples.

### Gene expression analysis

Gene expression was measured using the Nanostring nCounter Human Inflammation Panel (NanoString Technologies, Seattle, WA, USA). Probe hybridization, washing and counting were performed according to NanoString standard protocols (https://www.nanostring.com/support/product-support/support-documentation). Purified RNA (5 μL) was hybridized with the probe master stocks at 65°C for 24 h. Bound target and probe was then captured and excess probe washed away using the nCounter Prep Station and an automated protocol. Probe bound to target was then counted for each sample using the NanoString nCounter Digital Analyser.

### Statistical analysis

Gene expression data was analyzed using nSolver™ v4.0 (NanoString Technologies, Seattle, WA, USA) which enabled quality control, normalization, differential gene expression and pathway analysis. Background values were subtracted from raw data and normalized using internal positive control genes and 6 housekeeping genes (CLTC, GAPDH, GUSB, HPRT1, PGK1 and TUBB). Gene expression ratios were determined relative to uninfected day 0 cells and differential gene expression between virus strains compared using a t-test. Volcano plots with replicate samples were created using nSolver based on p values adjusted for false discovery rate using the method of Benjamini & Yekutieli (BY). Heat map representations of data were clustered based on Euclidean distance and principal component analyses (PCA) were made using ClustVis (https://biit.cs.ut.ee/clustvis/). Pathway scores were calculated using an algorithm incorporated in the nSolver v4.0 package [16].

## Results

### Detection of gene expression in WNV infected human neuroblastoma cells

**Fig. 1:**
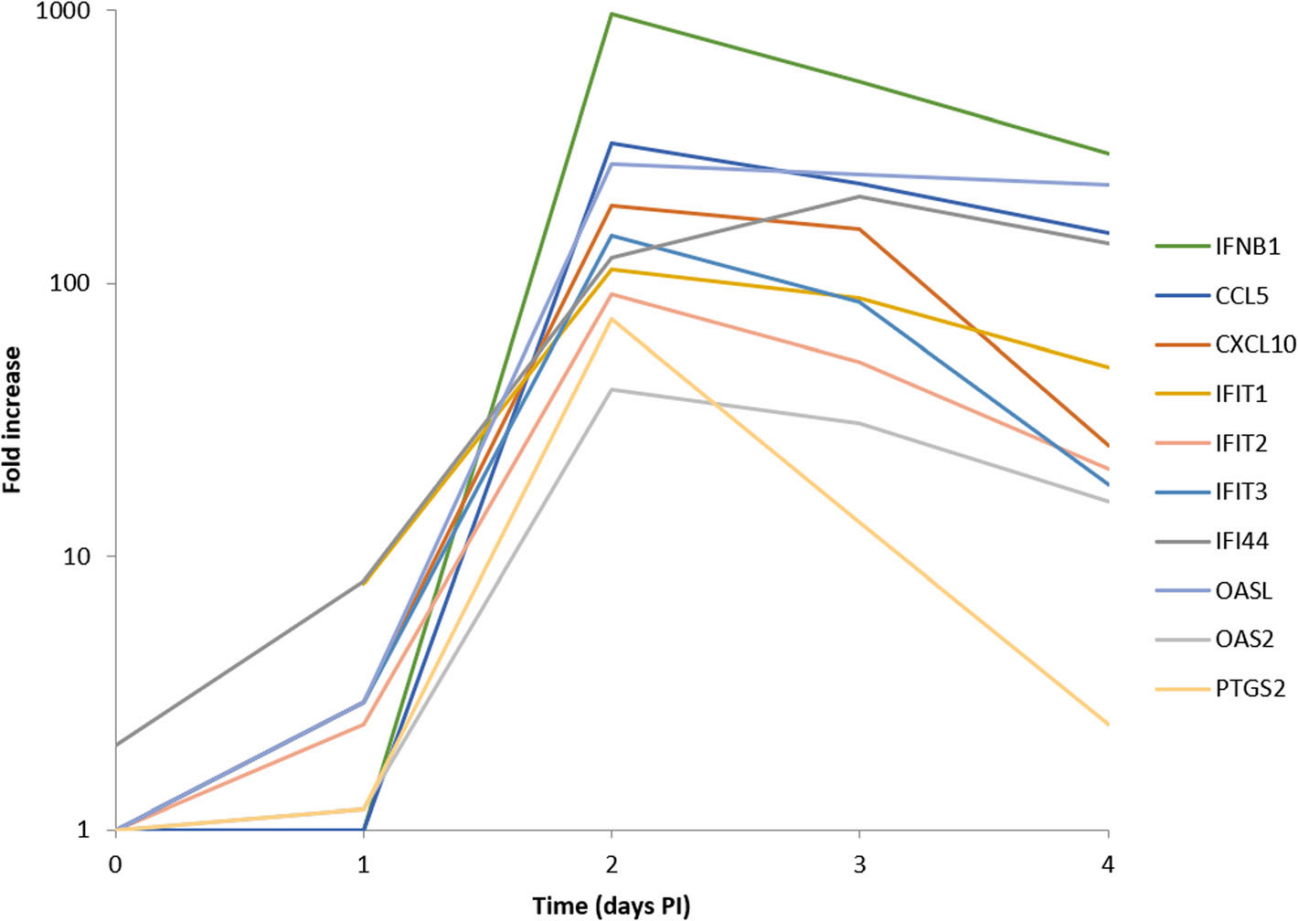
Time-course of gene induction by WNV (MRM16) infection. Gene expression of WNV infected cells relative to uninfected cells (fold induction) over a time-course (days 0–4 p.i.). The 10 highest WNV_KUN_ induced genes on the panel are shown

To determine the optimum time post-infection for induction of inflammation associated genes in infected neuroblastoma cells (SK-N-SH line), the cells were infected with the prototype WNV_KUN_ strain (MRM16) and the cells harvested at different time points post-infection (days 0, 1, 2, 3 and 4). Quantitative RT-PCR confirmed that the cells were infected (Additional file 1). Gene expression of 249 inflammation associated genes was measured using a nCounter panel (NanoString). To test that the cells were capable of responding to immuno-stimulants, poly(I:C) and LPS were used and gene expression of innate immune responses measured (Additional file 3). Poly(I:C) induced gene expression more strongly (> 10-fold induction: C1S, CCL5, CFB, CXCL9, IFI44, IFIT1, IFIT3, MX1, OAS2 and OASL) than LPS (> 10-fold induction: IL8) at the respective concentrations used. WNV_KUN_ (MRM16) infected cells produced strong inflammation gene expression relative to uninfected cells at the same time points. The expression of the 10 most strongly-induced genes (IFNB1, CCL5, OASL, CXCL10, IFIT3, IFI44, IFIT1, IFIT2, PTGS2 and OAS2) are presented as a line-plot (Fig. 1 and Additional file 3). These genes have all previously been shown to be induced by WNV infection [17–20] indicating an authentic response. The expression of these genes was maximal at day 2 post-infection; therefore, in subsequent experiments cells were harvested at this time-point.

**Fig. 2:**
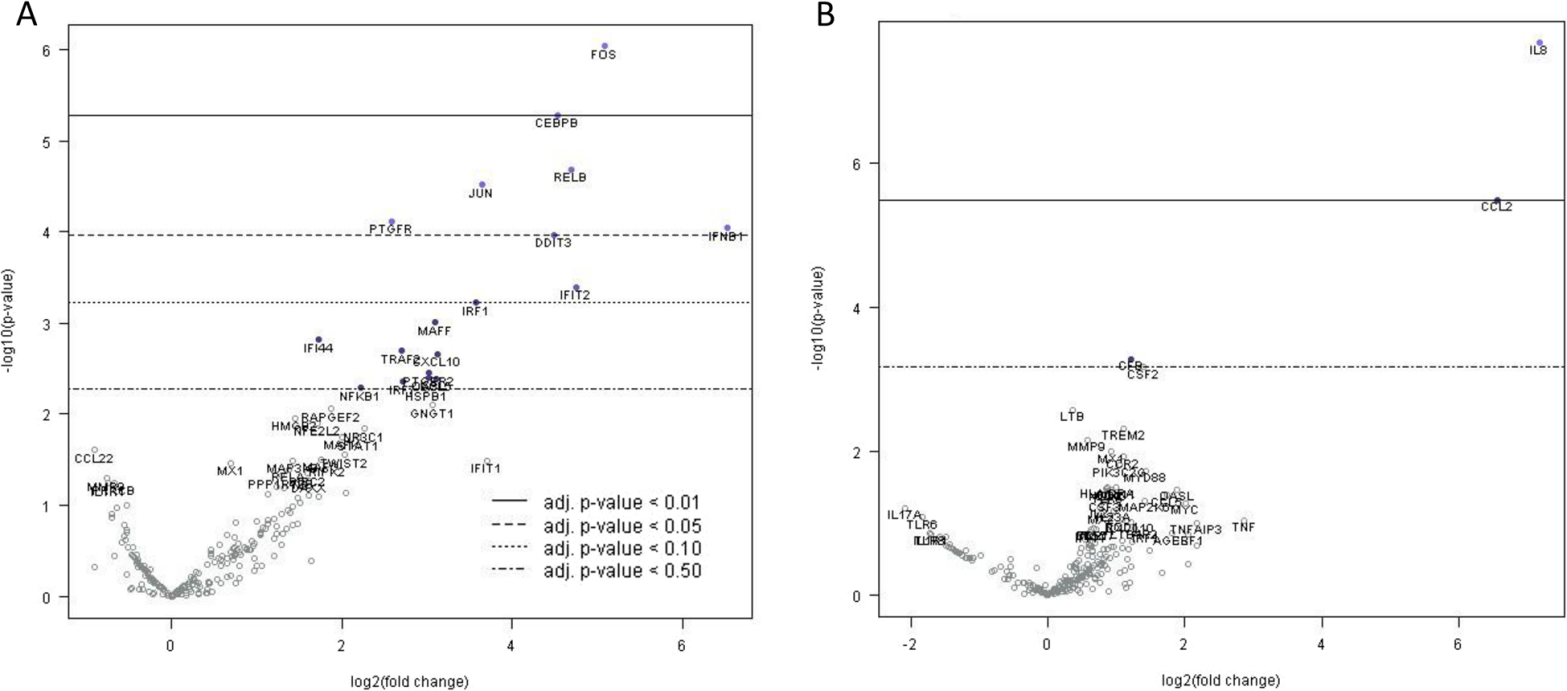
Induction of genes by infection with isolates of WNV_KUN_. **a** Gene expression of SK-N-SH cells infected with all isolates of WNV_KUN_ relative to uninfected cells as a baseline **b** Gene expression of SK-N-SH cells infected with the NSW2012 relative to WNV_KUN_ isolates (except Boort) as a baseline. Gene induction is presented as a volcano plot with Benjamini & Yekutieli adjusted probability values along the y-axis and fold induction along the x-axis

To determine the response to WNV_KUN_, SK-N-SH cells were infected with 10 different isolates in separate culture wells (Additional file 2: Table S1). These included two virulent isolates which included one isolated from a horse in 1984 (Boort), and another collected from mosquitoes which was closely related to the strain which caused an outbreak in Australian horses in 2011 (NSW2012) [7]. An isolate of Murray Valley encephalitis virus (MVEV) was included to enable a comparison with another Australian pathogenic flavivirus. Gene induction was measured for each isolate at the 48 h optimized time-point post-infection relative to uninfected cells. To measure changes in gene expression, infected cells were compared to uninfected controls. For those genes included on the panel, the response to WNV_KUN_ infection was one of induction rather than down-regulation. The expression of some genes was reduced but these were not statistically significant. Seven genes (FOS, CEBPB, RELB, JUN, PTGFR, IFNB1 and DDIT3; Fig. 2a) were determined to be significantly induced (BY adjusted *p*-value < 0.05). The most strongly-induced gene was FOS, also referred to as the proto-oncogene *c-fos*. The gene product forms a heterodimer with the product of another proto-oncogene JUN, or *c-jun*, in the activator protein complex 1 (AP-1). Interestingly, JUN was also strongly induced in WNV_KUN_ infected SK-N-SH cells. AP-1 has been linked to activation of apoptosis; therefore, induced FOS and JUN expression might also indicate initiation of programmed cell death by WNV infection. Related to this, another induced gene, DNA damage-inducible transcript 3 (DDIT3), is a pro-apoptotic protein which is induced in primary human cell cultures by WNV infection [20]. DDIT3 can interact with FOS and JUN [21], hence its induction further suggests activation of apoptosis by infection.

At least two other transcription factors were significantly induced by WNV_KUN_ infection (Fig. 2a). The first, CCAAT/enhancer-binding protein beta (CEBPB), is a transcription factor which can regulate a large number of genes including *c-fos* and cytokines including IL6, 8 and 12, and TNF-α (reviewed in [22]), and was previously shown to be upregulated in the brains of WNV infected mice [19, 23]. CEBPB is known to interact with DDIT3 [24], suggesting that they may be part of an apoptotic protein complex (or complexes) which also includes FOS and JUN. The second, RELB, is a member of a sub-family of the NF-κB transcription factors, which can itself form a complex with NF-κB [25]. NF-κB is downstream of the RIG-1 and TLR3/7 pattern recognition receptor (PRR) activated pathways, and is imported into the nucleus to activate IFNα/β and interferon stimulate genes (ISGs) in infected cells. IFNB1 (IFNβ gene) was also significantly and strongly induced by WNV_KUN_ infection consistent with PRR pathway activation. Finally, prostaglandin F receptor (PTGFR), which has several functions in the cell was induced, but its relevance to WNV infection is currently unclear.

**Fig. 3:**
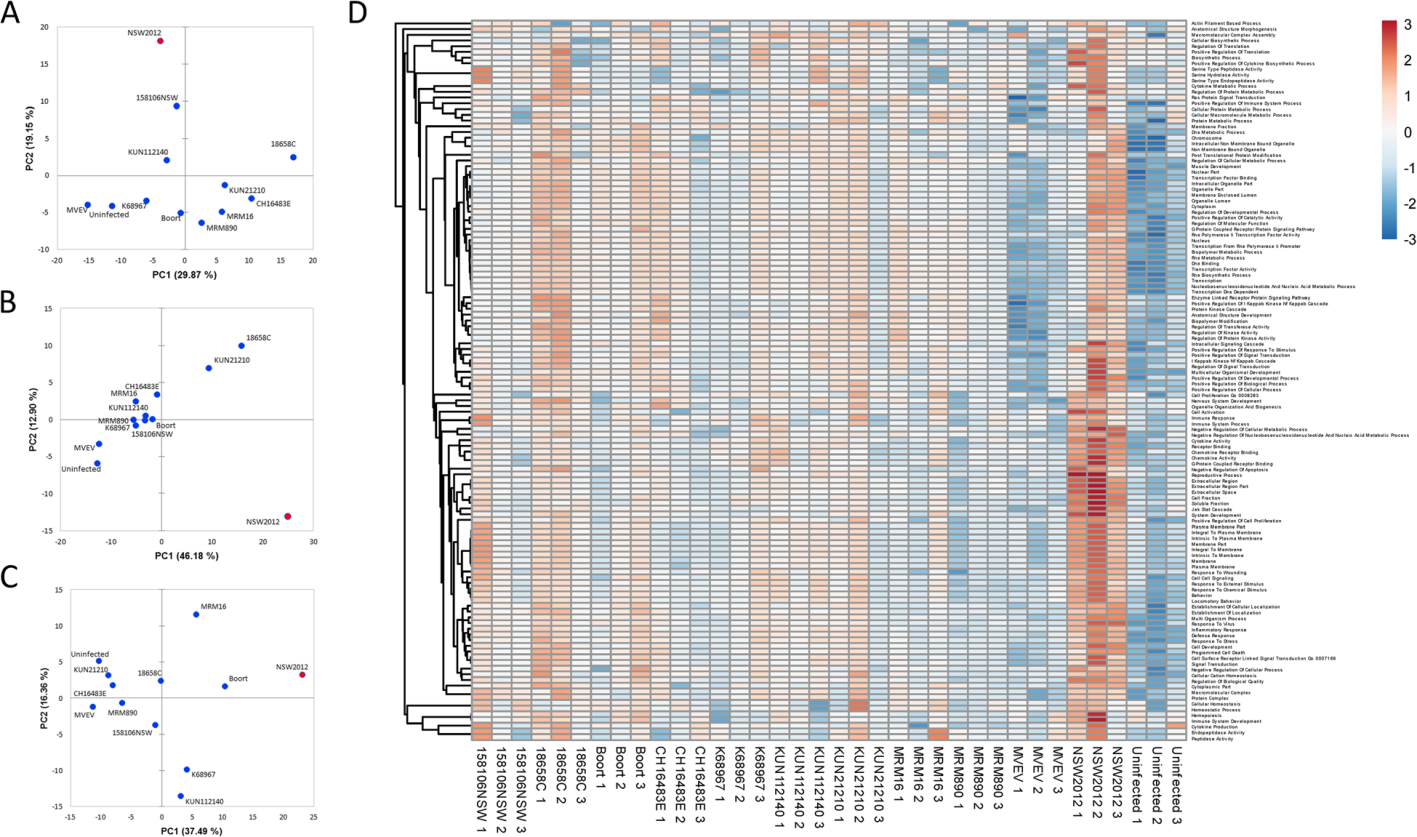
Analysis of WNV_KUN_ induced gene expression in SK-N-SH cells. **a**-**c** Principal component analysis plots of gene expression of the infected cells in two dimensions (3 experiments are shown). Isolates are represented as blue dots with the exception of NSW2012 which is represented as a red dot. **d** Pathway analysis of the triplicate WNV_KUN_ infections. Illustrates pathway score over a > 6 log_2_ range (> 64-fold) shown arbitrarily on the scale from − 3 to 3. Gene expression is represented in the range from lower (blue) to higher (red)

To examine the innate immune gene induction response to WNV_KUN_ infection, the 249 inflammation-associated genes on the panel were analyzed using principal component analysis (PCA). The results of the three separate infections showing variation in gene induction across two axes are shown (Fig. 3a-c). The plots of isolates varied among experiments, with no distinct clustering. The position of the virulent NSW2012 isolate was consistently distant relative to the positions of the other isolates in all three experiments. However, that was not the case for the virulent Boort isolate suggesting the nature of its virulence may be different to NSW2012. Gene induction by NSW2012 infection was then compared with the isolates (excluding Boort). Two genes, IL8 and the chemokine CCL2 (also known as MCP-1), were clearly significantly differentially induced (Fig. 2b). When gene induction of the Boort infected cells was compared with gene inductions resulting from infections with other WNV_KUN_ isolates (excluding NSW2012), no significant gene expression differences were observed (Additional file 4: Figure S1). Overall, the above findings suggested that the expression of the induced genes may be different for the NSW2012 isolate compared to the others, and may possibly relate to its greater virulence.

To further explore the possibility that the differential gene induction may reveal markers of virulence, a pathway analysis was conducted. Scores were calculated for 126 different inflammation-associated pathways for the SK-N-SH cells infected with the WNV_KUN_ isolates and plotted as a heat-map (Fig. 3d and Additional file 3). Between the three separate infections, WNV_KUN_ infected cells generally had higher pathway scores than uninfected cells indicative of virus infection. NSW2012 demonstrated the highest and most consistent (all three experiments) pathway scores among the isolates including pathways Iκ-B kinase/NF-κB cascade, cytokine activity, chemokine activity, G-protein coupled receptor binding, JAK-STAT cascade, response to virus, inflammatory response, and programmed cell death. Strain 18658C showed higher pathway scores than the other WNV_KUN_ isolates, albeit with lower scores than NSW2012, in two out of three experiments. Collectively, the above data suggest that the NSW2012 isolate has a more inflammatory phenotype than the other isolates when infecting the neuroblastoma cell line SK-N-SH. By contrast, cells infected with MVEV did not show statistically significant induction of individual genes (Additional file 4: Figure S1). In addition, MVEV showed consistently generally lower pathway scores than WNV_KUN_. This may have been due to different growth kinetics for MVEV replication, as the assay was optimized for WNV_KUN_, resulting in gene induction kinetics that were not directly comparable.

### Gene induction in WNV-infected, iPSC-derived neuronal cultures

Continuously cultured cell lines such as SK-N-SH have a more metabolically active cellular environment than primary cell cultures or cells from tissue. This would be expected to influence signaling pathways and, as a result, gene expression. Hence, infection experiments were conducted in neuronal cultures derived from human induced pluripotent stem cell (iPSC) derived brain organoids to determine whether the phenotypic differences observed between isolates in the SK-N-SH cells could be recapitulated in this cell system. A smaller subset of WNV_KUN_ isolates was used in these experiments and included isolates MRM16 and K68967, the virulent isolates Boort and NSW2012, and MVEV for comparison. Cells were harvested at 48 h post-infection to be consistent with earlier experiments.

**Fig. 4:**
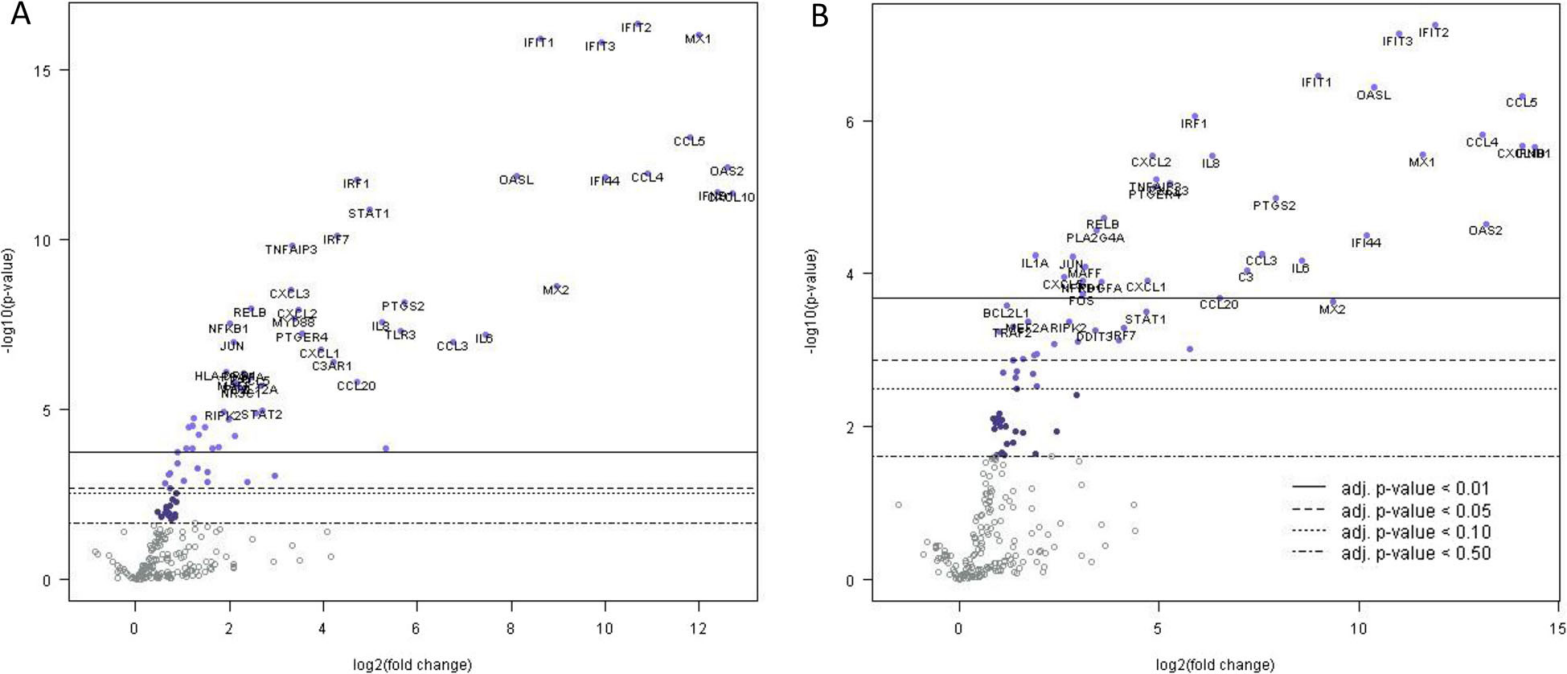
Induction of genes by infection. **a** Gene expression of neuronal cells infected with all isolates of WNV_KUN_ relative to uninfected cells as a baseline **b** Gene expression of neuronal cells infected with MVEV relative to uninfected cells as a baseline. Gene induction is presented as a volcano plot with Benjamini & Yekutieli adjusted probability values along the y-axis and fold induction along the x-axis

As the neuronal cultures were stem-cell derived, it was firstly necessary to determine if they were permissive to WNV_KUN_ infection and replication. Four separate infections were performed, and infected neuronal cells were found to have significantly lower cycle threshold values by qRT-PCR assay, with ΔC_T_ values (day 0 – day 2 post-infection) typically > 9 (Additional file 1) compared to non-infected cells. Hence, the cells were permissive to WNV_KUN_ (and MVEV) infection. RNA from the cells was then used to measure gene induction as previously. Similarly to SK-N-SH cells, infection resulted primarily in induction (as opposed to down-regulation) of those genes included on the panel, for both WNV_KUN_ and MVEV (Fig. 4 and Additional file 5). There were more induced genes that had lower probability (i.e. statistically significant) scores in the neuronal cultures than the SK-N-SH cells. This may have been a result of the less variable baseline of expression in the uninfected cells. There were many induced genes shared in common between cells infected with these two viruses. Similarly to SK-N-SH cells, WNV_KUN_ infected cells showed induction of IFNB1, RELB, and JUN genes. Of these, only the latter suggested an apoptotic response. There were many other inflammation-associated genes that were found to have significantly higher induction than uninfected cells (Fig. 4a). They included members of the IFIT group, chemokines, cytokines, interferon regulatory factors, NF-κB, OASL, MyD88 and STAT genes. Interestingly, another gene associated with prostaglandin function (prostaglandin-endoperoxide synthase 2 or PTGS2) was also induced and, along with a similar observation in infected SK-N-SH cells, suggests that this gene may play a role in the innate response to WNV. In the neuronal cultures, the gene expression induced by infection with Boort and NSW2012 strains was not significantly different to that of the other strains (Additional file 4: Figure S1).

**Fig. 5:**
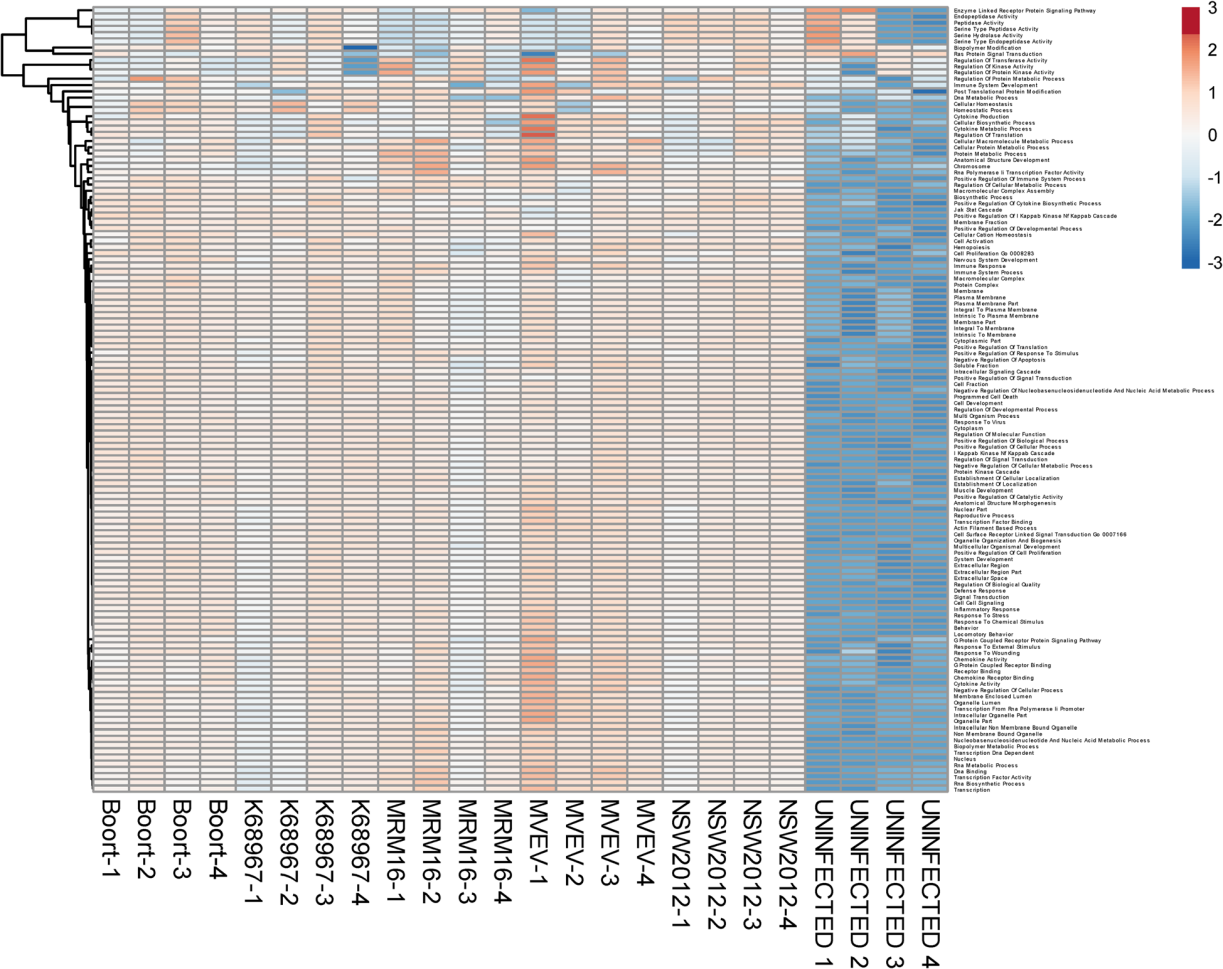
Analysis of WNV_KUN_ induced gene expression in iPSC-derived neuronal cells. Pathway analysis of WNV_KUN_ infections in quadruplicate. Illustrates pathway score over a > 6 log_2_ range (> 64-fold) shown arbitrarily on the scale from − 3 to 3. Gene expression is represented in the range from lower (blue) to higher (red)

Pathway scores were calculated using the gene induction data and are presented as a heat-map (Fig. 5 and Additional file 3). As with the SK-N-SH cells, uninfected neuronal cells had consistently lower pathway scores than infected cells. A characteristic of the neuronal cells was that gene induction in the WNV infected cells was less variable across isolates and replicates. However, there were no visibly obvious differences that were consistent among all 4 replicate infections between the more and less virulent WNV_KUN_ isolates. In contrast to the SK-N-SH cell infections, MVEV showed a more activated phenotype having consistently higher scores in such pathways as G protein coupled receptor protein signaling pathway, G couple protein receptor binding, chemokine activity, chemokine receptor binding, and cytokine activity. The above data show that the cell environment is an important determinant in the innate immune response to viruses.

## Discussion

WNV infection can cause a range of symptoms from asymptomatic to lethal encephalitis. The determinants of the disease outcome are likely a result of the complex interplay between the virus and the immune response of the host; however, this is incompletely understood. To explore this area, we used a quantitative immune gene expression assay to measure the induction of a panel of 249 inflammation-associated genes to determine which are induced in the host during WNV_KUN_ infection, how this leads to activation of innate pathways, and whether specific genes were induced by infection with more pathogenic strains. We found that as with other strains of WNV, specific pathways were activated, and two genes were significantly more induced during infection with the virulent NSW2012 isolate.

The induced gene FOS is one of the immediate early genes (IEGs), which are those genes induced in neurons by external stimuli without requiring de novo protein synthesis. FOS is known to be expressed in neuronal and other brain cultures, and in mouse and human brains subject to various stimuli such physical and chemical insults, heat shock, osmotic stimulation, various drugs, and induced seizures (reviewed in [26]). It is also associated with transformation of some cell lines and cancer [27]. WNV_KUN_ infection of SK-N-SH cells strongly suggested the activation of an apoptotic response as indicated by induction of AP-1 (i.e. FOS and JUN) and DDIT3. Flavivirus infection in general is known to induce apoptosis, and specifically in the case of WNV infection of mice [28], neuronal cell lines such as Neuro 2a, neurons derived from ES cells, and SK-N-MC cells (human neuroepithelioma) (reviewed in [29]). WNV proteins C, M, NS2A and NS3 are pro-apoptotic [29]. Hence, the induction of genes related to apoptosis is consistent with previous observations of WNV and other flaviviruses. However, the many observations of FOS and JUN expression in neural cells and tissue after exposure to multiple stimuli suggests there may be other functions for their gene products which are unrelated to apoptosis. Hence, our observations of increased FOS expression may not directly relate to pathogenesis during infection in vivo.

Whilst induction of apoptosis was more apparent in the neuroblastoma cell line, the expression of prostaglandin and the respective receptor-related genes was common to both cell systems. In the SK-N-SH cell, the prostaglandin receptor gene PTGFR was induced. In the iPSC-derived neuronal cultures, the PTGS2 gene (also known as COX-2) was induced. The latter gene encodes for the enzyme responsible for converting arachidonic acid to prostaglandin H2, an important inflammatory mediator. In addition, the PTGER4 gene was induced which codes for a receptor that binds prostaglandin E2. Interestingly, prostaglandin E2 is an activator of the Wnt signaling pathway that regulates interferon response in WNV infection [30]. Overall, these observations suggest a role for prostaglandins and their receptors in innate responses to WNV infection. Prostaglandins are produced in a wide range of tissues including neurons where they contribute to nociception [31]. In fact, COX-2 is a major target for analgesic therapeutics. Future research should determine whether prostaglandin-related signaling is tissue-specific (i.e. neurons), or part of a broader response to controlling virus infection.

As expected, there were differences in the responses of the two cell systems used in this study. Both resulted in a significant increase in the induction of inflammation associated genes. However, the gene induction in the neuronal cells was less variable, possibly due to the lower growth rate and generally less active state of these cells relative to the SK-N-SH cell line. This phenomenon, may also be related to the observation that the NSW2012 infection of the SK-N-SH cells showed stronger induction of two genes (CCL2 and IL8), but this was not observed in the neuronal line. It may be that the constitutively active state of this cell line, in combination with the more virulent genotype of NSW2012, resulted in the activation of signaling pathways. In contrast the Boort isolate, which has an intermediate virulence between NSW2012 and the prototype strain [7], did not show statistically significant gene induction specific to this isolate. Hence, the virulence of this isolate may involve the activation of different pathways, or perhaps genome changes that occurred during its unknown passage history [7] have altered its phenotype. The other flavivirus included in this study, MVEV, also induced similar genes to those induced by WNV_KUN_; however, their expression patterns would not be directly comparable as the two viruses had different growth kinetics.

Important aspects of the neurotropism of a virus are both its neurovirulence and neuro-invasiveness. The latter is those characteristics of a virus which give it the ability to access the brain parenchyma. In one popular hypothesis, host innate response leading to pro-inflammatory cytokine and chemokine production are thought to permeabilize the blood-brain barrier (BBB), enabling viruses to infect the brain [10, 32, 33]. Proteins linked to BBB permeabilization include TNFα, IL-6, IL-1β. IFNγ, and matrix metalloproteinases (MMPs). In the case of NSW2012, TNF-α was induced (Fig. 2b) in comparison with the other WNV_KUN_ isolates but this was not statistically significant. The two proteins that were specifically more induced were pro-inflammatory IL-8 and CCL2. IL-8 has previously been shown to be upregulated in WNV infected primary human cultures [20] and cell lines [34], and has been measured at higher levels in patients with encephalitis of infectious aetiology [35]. CCL2 has previously been shown to be induced in WNV infected patients [36], primary human cell cultures [17] and mice [18, 19, 23, 28]. CCL2 has been linked to infiltration of monocytes and leukocytes into the brain during virus infection. In WNV infection, CCL2 is also required for efficient monocyte infiltration in mouse brain [37]. Evidence for this cytokine being a general host marker for pathogenic virus infection is the observation that CCL2 expression was differentially upregulated in the brains of mice infected with a more neurovirulent strain of Venezuelan equine encephalitis virus in comparison with a less virulent strain [38]. Furthermore, CCL2 drove monocyte infiltration into the brains of mice acutely infected with Theiler’s murine encephalomyelitis virus [39], and HIV-1 infection resulted in CCL2 mediated leukocytes across the BBB in a tissue culture model [40]. Hence CCL2 may be an important immune-mediated pathology. Both these proteins, along with the others induced by infection, could have contributed to induce a host response which was conducive to BBB permeabilization and, consequently, brain infection. These in vitro observations provide the basis for future animal experiments which will elucidate the immuno-pathology of IL-8 and CCL2 in vivo.

## Conclusion

In this study we have measured host cell innate immune responses to WNV_KUN_ infection using two different cell systems. The neuroblastoma cell line SK-N-SH showed itself to be a useful system for elucidating the mechanism of virulence of the more pathogenic NSW2012 isolate. Using this in vitro cell system, two genes were identified that were more strongly induced during infection with a virulent strain (IL-8 and CCL2), and these are strong candidates for further in vivo studies. The iPSC derived neuronal system was found to have less variable gene induction which resulted in the determination of the broad gene induction response to WNV_KUN_. Both systems will find future application to understanding virus pathogenicity, and ultimately for the establishment of early warning systems for the detection of virulent emerging viruses.

## Supplementary information

**Additional file 1.** Quantitative RT-PCR on cell infections. Example data for fluorescence curves and cycle threshold values (C_T_) values are given for infected cells at day 0 and day 2 post-infection. Data for MVEV and WNV_KUN_ infections are shown.

**Additional file 2: Table S1.** Information on the isolates used in these experiments.

**Additional file 3.** nCounter panel data. (A) Time course of infection experiments in SK-N-SH cells given as a ratio relative to uninfected cells (day 0). Pathway scores used for heat-map generated from nCounter panels of infected (B) SK-N-SH and (C) neuronal cells.

**Additional file 4: Figure S1.** Volcano plots of gene expression analysis for additional virus infections performed in this study. (A) Gene induction of Boort isolate infected SK-N-SH cells using WNV_KUN_ isolates (except NSW2012) infected cells as a baseline. (B) Gene induction of MVEV infected SK-N-SH cells using uninfected cells as a baseline. (C) Gene induction of Boort isolate infected neuronal cells using WNV_KUN_ isolates (except NSW2012) infected cells as a baseline. (D) Gene induction of NSW2012 isolate infected neuronal cells with WNV_KUN_ isolates (except Boort) infected cells as a baseline. The B-Y adjusted probability is show on the vertical axis and fold induction is shown along the horizontal axis.

**Additional file 5.** Differential analysis data.

## Data Availability

All data generated or analyzed during this study are included in this published article and its Additional files.

## Abbreviations

AP-1: Activator protein complex 1
BBB: Blood-brain barrier
BY: Benjamini & Yekutieli
CEBPB: CCAAT/enhancer-binding protein beta
DDIT3: DNA damage-inducible transcript 3
DMEM: Dulbecco’s Modified Eagles Medium
IEGs: Immediate early genes
iPSCs: Induced pluripotent stem cells
ISGs: Interferon stimulate genes
MVEV: Murray Valley encephalitis virus
PAMP: Pathogen-associated molecular pattern
PCA: Principal component analyses
PRRs: Pattern recognition receptors
PTGFR: Prostaglandin F receptor
qRT-PCR: Quantitative reverse-transcription polymerase chain reaction
SK-N-SH cells: Neuroblastoma cell line
WNV: West Nile virus
