# Supplementary material for: Inflammatory responses to a pathogenic West Nile virus strain
Source: BMC Infect Dis. 2019 Oct 29;19:912. doi: 10.1186/s12879-019-4471-8 (PMC6819652; doi:10.1186/s12879-019-4471-8)
Supplement: Supplementary file 1 — Additional file 1. Quantitative RT-PCR on cell infections. Example data for fluorescence curves and cycle threshold values (CT) values are given for infected cells at day 0 and day 2 post-infection. Data for MVEV and WNVKUN infections are shown. [file 12879_2019_4471_MOESM1_ESM.docx]

**Additional file 1: Quantitative RT-PCR confirmation of cell infection**

Infection of SK-N-SH cells with WNV_KUN_:


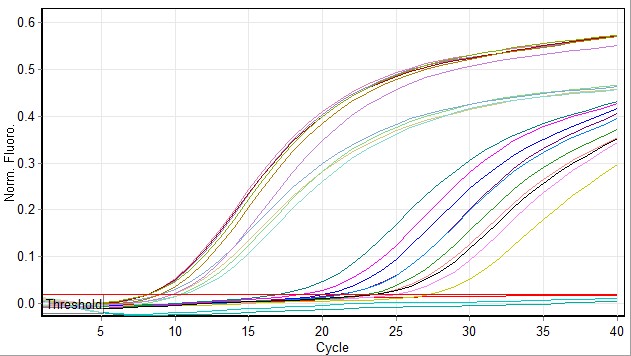


| Name | Ct of Day 0 | Ct of Day 2 |
| --- | --- | --- |
| Uninfected |  |  |
| MRM890 | 27.34 | 9.92 |
| MRM16 | 20.27 | 10.48 |
| CH16483E | 21.34 | 10.61 |
| KUN21210 | 25.05 | 9.16 |
| Boort | 21.62 | 8.28 |
| 18658C | 17.23 | 9.95 |
| KUN112140 | 23.61 | 8.22 |
| K68967 | 23.16 | 8.27 |
| 158106NSW | 18.65 | 9.02 |
| NSW2012 | 23.66 | 8.27 |
| Kunjin-NTC |  |  |

Infection of SK-N-SH cells with MVEV:


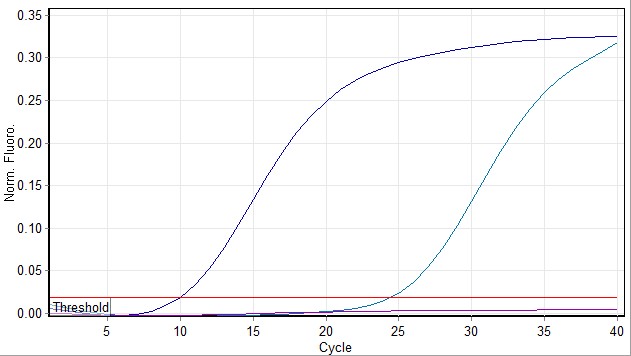


| Name | Ct |
| --- | --- |
| MVEV Day 0 | 24.55 |
| MVEV Day 2 | 9.99 |
| MVEV-NTC |  |

Infection of neuronal cells with WNV_KUN_:


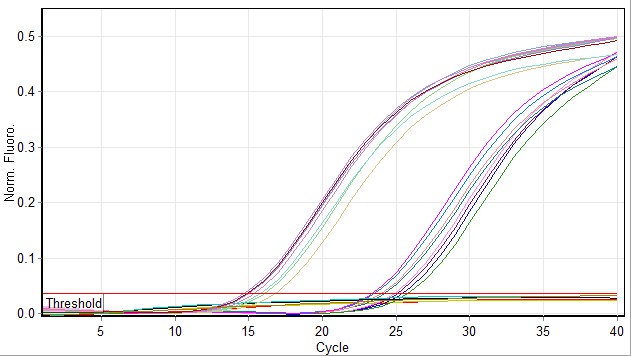


| Name | Duplicate | Ct of Day 0 | Ct of Day 2 |
| --- | --- | --- | --- |
| Kun-Uninfected | Sample-1 |  |  |
| Kun-Uninfected | Sample-2 |  |  |
| Kun-MRM16 | Sample-1 | 25.46 | 16.85 |
| Kun-MRM16 | Sample-2 | 25.11 | 16.1 |
| Kun-Boort | Sample-1 | 24.9 | 15.84 |
| Kun-Boort | Sample-2 | 23.58 | 15.07 |
| Kun-K68967 | Sample-1 | 24.25 | 15.04 |
| Kun-K68967 | Sample-2 | 24.07 | 14.77 |
| Kun-NSW2012 | Sample-1 | 25.81 | 15.41 |
| Kun-NSW2012 | Sample-2 | 23.43 | 14.97 |
| Kun-NTC |  |  |  |

Infection of neuronal cells with MVEV:


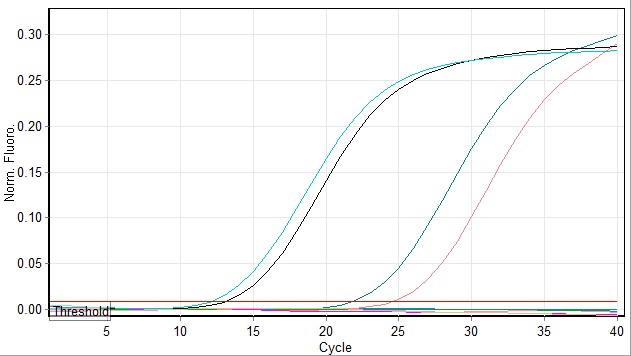


| Name | Duplicate | Ct of Day 0 | Ct of Day 2 |
| --- | --- | --- | --- |
| MVEV-Uninfected | Sample 1 |  |  |
| MVEV-Uninfected | Sample 2 |  |  |
| MVEV-MVEV | Sample 1 | 21.91 | 13.2 |
| MVEV-MVEV | Sample 2 | 24.69 | 12.17 |
| MVEV-NTC |  |  |  |
