# Supplementary material for: Inflammatory responses to a pathogenic West Nile virus strain
Source: BMC Infect Dis. 2019 Oct 29;19:912. doi: 10.1186/s12879-019-4471-8 (PMC6819652; doi:10.1186/s12879-019-4471-8)
Supplement: Supplementary file 2 — Additional file 2: Table S1. Information on the isolates used in these experiments. [file 12879_2019_4471_MOESM2_ESM.docx]

**Additional file 2: Table S1. Isolates used in this study**

| **Isolate** | **Year** | **State collected** | **Species** | **Reference** | **Accession number** |
| --- | --- | --- | --- | --- | --- |
| 18658C | 1975 | Unknown | - | - | KX394381 |
| Boort | 1984 | Victoria | *Equus caballus* (horse) | Prow *et al*, 2016 | KT934796 |
| K68967 | 2009 | Western Australia | *Cx. annulirostris* | Prow *et al*, 2016 | KT934802 |
| MRM16 | 1960 | Queensland | *Cx. annulirostris* | Doherty, 1963 | KX394396 |
| MRM890 | 1963 | Queensland | *An. farauti* | Doherty, 1968 | KX394407 |
| CH16483E | 1974 | Queensland | *Cx. annulirostris* | QH records | KX394387 |
| KUN112140 | 1999 | New South Wales | *Cx. annulirostris* | QH records | KX394395 |
| KUN21210 | 1978 | Western Australia | *Cx. annulirostris* | QH records | KX394394 |
| 158106NSW | 2011 | New South Wales | *Cx. annulirostris* | QH records | KX394382 |
| NSW2012 | 2012 | New South Wales | *Cx. annulirostris* | Prow *et al*, 2016 | KT934804 |
| MVEV (MRM66) | 1960 | Queensland | *Cx. annulirostris* | Doherty, 1963 | - |

QH, Queensland Health; - , no records.
