# Supplementary figures and images for: Inflammatory responses to a pathogenic West Nile virus strain
Source: BMC Infect Dis. 2019 Oct 29;19:912. doi: 10.1186/s12879-019-4471-8 (PMC6819652; doi:10.1186/s12879-019-4471-8)

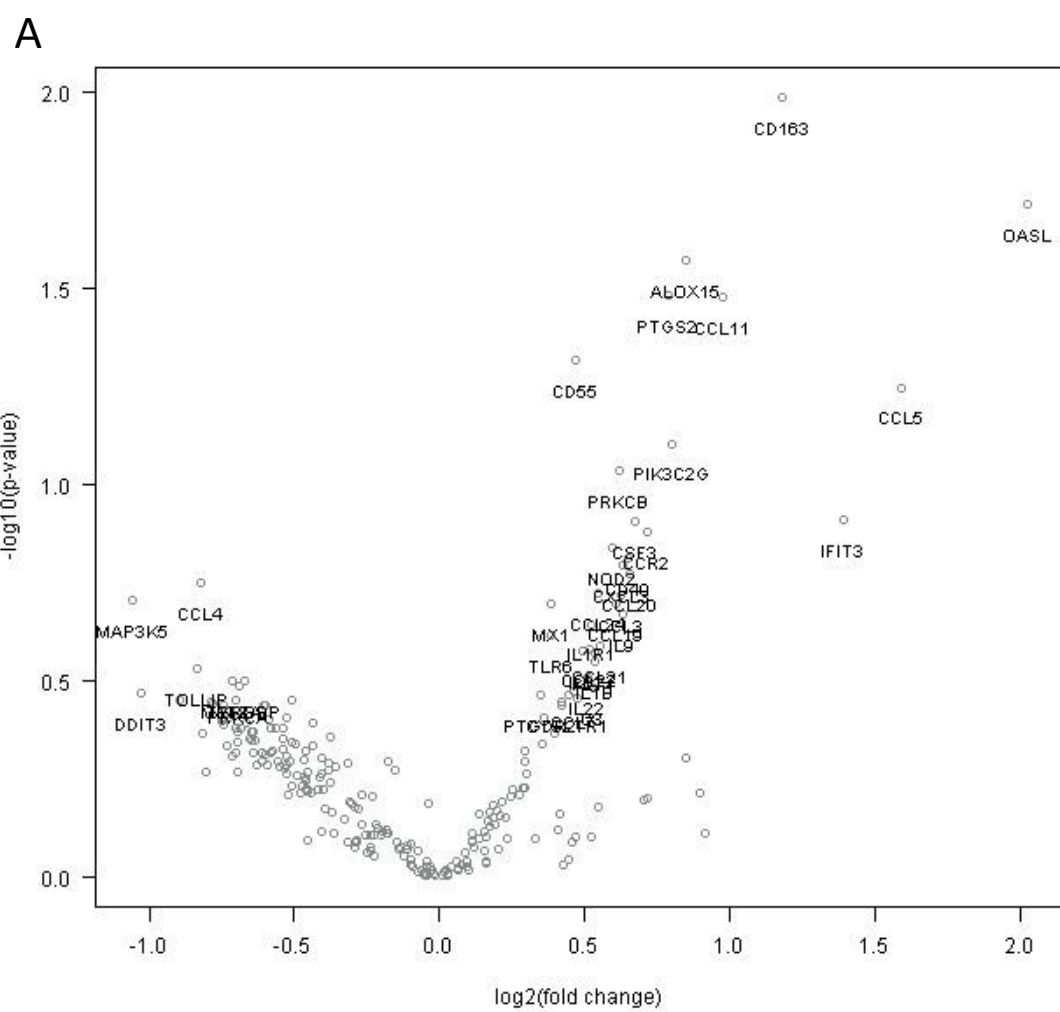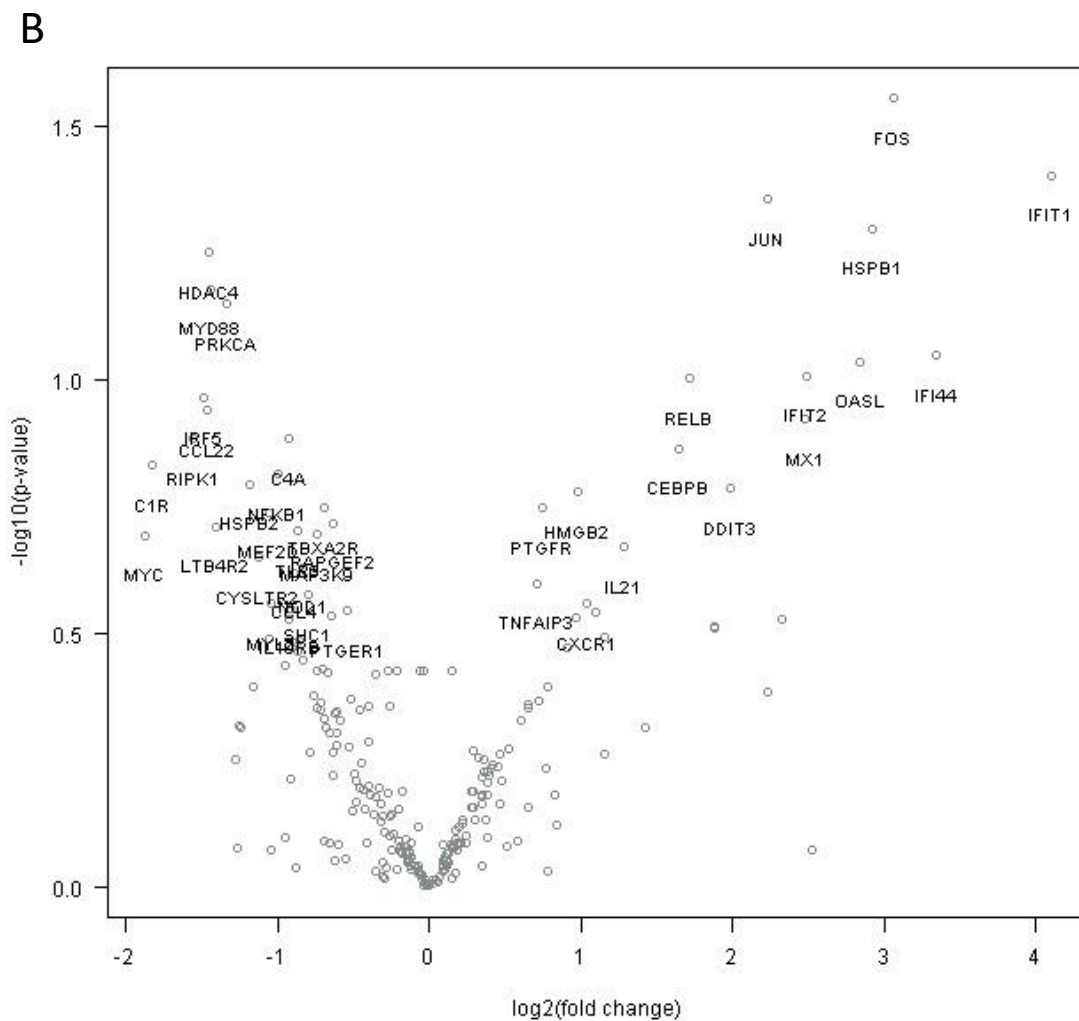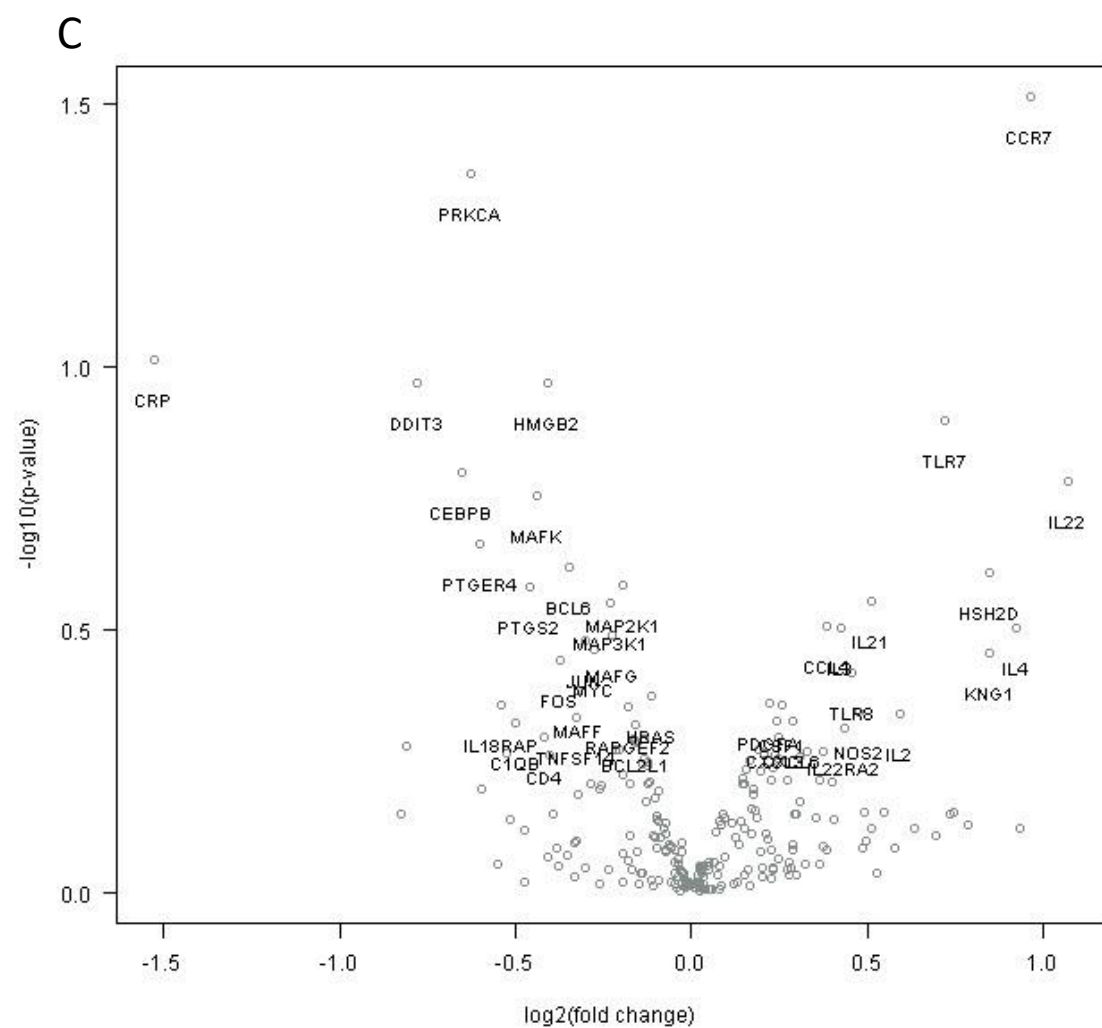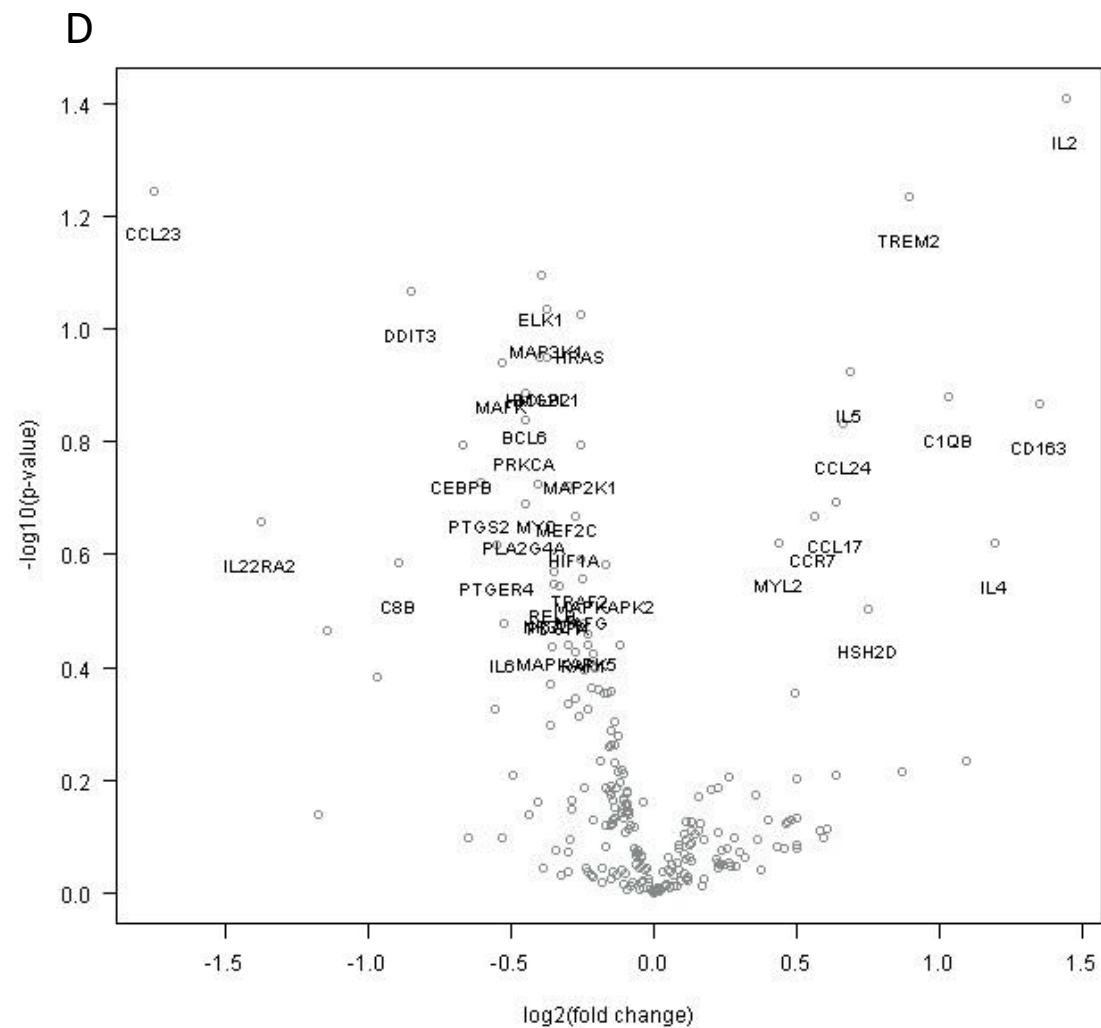

Supplement: Supplementary file 4 — Additional file 4: Figure S1. Volcano plots of gene expression analysis for additional virus infections performed in this study. (A) Gene induction of Boort isolate infected SK-N-SH cells using WNVKUN isolates (except NSW2012) infected cells as a baseline. (B) Gene induction of MVEV infected SK-N-SH cells using uninfected cells as a baseline. (C) Gene induction of Boort isolate infected neuronal cells using WNVKUN isolates (except NSW2012) infected cells as a baseline. (D) Gene induction of NSW2012 isolate infected neuronal cells with WNVKUN isolates (except Boort) infected cells as a baseline. The B-Y adjusted probability is show on the vertical axis and fold induction is shown along the horizontal axis. [file 12879_2019_4471_MOESM4_ESM.pdf]
